# Hare-Lip and Cleft Palate

**Published:** 1889-09

**Authors:** 


### Hare-Lip and Cleft Palate.

Here is a case that tells you the whole story. You will see at once that it is hare-lip, and that it came very near being double. In addition, there is a deficiency in the palate on each side, leaving quite a prominent intermaxillary bone in the middle. The union is complete behind. These cases of course call for operation. The hare-lip should be operated upon first, because the bringing together of the lip has some controlling action over the gap in the roof of the mouth, so that when the staphylorrhaphy is performed when the child is five or six years old, there will then be a much smaller chink in the palate to close up. The time for operation is a question about which surgeons are a little unsettled; but the best time is when the child is two or three months old, unless there is some pressing need of operating earlier.

The operation consists simply in freshening the edges, bringing them together, and liberating the lip from its attachment to the mucous membrane covering the jaw. Some surgeons recommend an elliptical, others an angular incision of the lip, the object being to avoid a little depression that is likely to occur just at the point of union at the free border of the lip. In this case the left intermaxillary bone projects forward, and by pressing against the lip may prevent its uniting. Sometimes the prominent portion of the bone has to be removed so as to allow the cut surfaces to be brought together.

A German Odontological Society has recently been organized in Berlin under the presidency of Professor Busch. It will hold a general meeting and five scientific sittings every year.

Exercise and Medicine.—Boerhaave, the famous physician, declared that a man was more likely to get well by climbing a tree than by drinking a decoction made of its leaves! that is, he thought exercise better than medicine. It is on this principle that the Queen of Sweden, whose nervous condition has given rise to much anxiety, is being treated. She is ordered to make her bed and sweep her room, besides taking a large amount of walking exercise. This method—the "housemaid treatment," as he calls it—has inspired a cynical journalist with some suggestions which are, perhaps, wiser than he knows. He advises the "office-boy treatment" for the dyspeptic millionaire, the "groom treatment" for the Crœsus whose liver is too much with him, the "country postman treatment" for the obese financier; the "nursemaid treatment" for the hysterical woman who can not stand a child's cry, and the "old clothes woman treatment" for the fine lady who faints at the sight of powder. Probably the "treatments" would be efficacious—if the patient would submit. —*London Hospital.*

